# A Promising Approach to Provide Appropriate Colon Target Drug Delivery Systems of Vancomycin HCL: Pharmaceutical and Microbiological Studies

**DOI:** 10.1155/2014/182197

**Published:** 2014-01-14

**Authors:** Kadria A. Elkhodairy, Samar A. Afifi, Azza S. Zakaria

**Affiliations:** ^1^Department of Pharmaceutics, College of Pharmacy, King Saud University, P.O. Box 22452, Riyadh 11495, Saudi Arabia; ^2^Department of Industrial Pharmacy, Faculty of Pharmacy, Alexandria University, Alexandria, Egypt; ^3^Department of Pharmaceutics, National Organization for Drug Control and Research, Giza, Egypt; ^4^Department of Microbiology, Faculty of Pharmacy, Alexandria University, Alexandria, Egypt

## Abstract

Vancomycin HCl was prepared as orally administered colon target drug delivery tablets for systemic therapy. Tablet matrices containing 10–60% of tablet weight of guar gum (F1–F6) were prepared by direct compression and subjected to *in vitro* release studies to explore their sustained release in the colon. Various synthetic and natural polymers were incorporated to F6 to modify the drug release rate. Different 15 matrix tablet formulations (F6–F20) were enteric coated with hydroxypropyl methyl cellulose phthalate. F6, F13 and F20 showed promising sustained release results having median dissolution time (MDT) values: 8.25, 7.97, and 7.64, respectively. Microbiological assay was performed to test the efficacy of F6, F13, and F20 to inhibit clinical *Staphylococcus aureus* (SA) isolates. Bactericidal activity of F6 was reached after 2, 4, and 24 hours of incubation against MSSA 18, MRSA 29, and MRSA 11 strains, respectively, while it was reached within 6–8 hours in case of F13, and F20 against all strains tested. F13 enhanced log microbial reduction by 1.74, 0.65 and 2.4 CFU/mL compared to F6 while it was 1, 2.57 and 1.57 compared to F20 against MSSA18, MRSA11 and MRSA29, respectively. Vancomycin HCl tablets displayed a promising sustained release *in vitro* and microbiological inhibitory action on all isolates tested.

## 1. Introduction

Colon target drug delivery system (CDDS) is highly desirable for local treatment of a variety of bowel diseases such as ulcerative colitis, Crohn's disease, amebiasis, colonic cancer, local treatment of colonic pathologies, and systemic delivery of protein and peptide drugs [1, 2].

The colon is believed to be a suitable absorption site for peptides and protein drugs for the following reasons: (i) less diversity and intensity of digestive enzymes, (ii) less proteolytic activity of colon mucosa leading to better protection from hydrolysis and enzymatic degradation in duodenum and jejunum, (iii) greater systemic bioavailability [3] and (iv) long colon residence time (5 days) and high responsiveness to absorption enhancers [4].

Vancomycin hydrochloride is amphoteric glycopeptide antimicrobial substance produced by the growth of certain strains of *Streptomyces orientalis *used in the treatment of enterocolitis caused by *Staphylococcus aureus* and antibiotic associated pseudomembranous colitis caused by *C. difficile *[5]. Vancomycin HCl being a peptide drug is a good candidate for colon targeted drug delivery.

*Staphylococcus aureus* has been one of the major causes of fatal nosocomial infections as well as community-associated infections [6]. Methicillin resistant *S. aureus* (MRSA) is important because, in addition to being methicillin resistant, most strains are also resistant to other *β*-lactam antibiotics, with the exception of glycopeptides antibiotics [7]. Because of the extensive occurrence of MRSA, methicillin was replaced by vancomycin to be the therapy of staphylococcal infections [8]. As the use of vancomycin was drastically increased, strains of *Staphylococcus aureus* and other species of staphylococci with decreased susceptibility to vancomycin and other glycopeptides were emerged. The Clinical Laboratory Standard Institute (CLSI) defines staphylococci which need vancomycin's concentration of less than or equal 2 *µ*g/mL to inhibit growth as “susceptible;” those need 4–8 *µ*g/mL for inhibition are considered “intermediate” and if the vancomycin's concentration needed for growth inhibition is more than 16 *µ*g/mL, then the staphylococci are considered “resistant” [9].

Vancomycin has been used for more than 60 years. Its mode of action is by blocking cell wall synthesis. Vancomycin binds with high affinity to the D-Ala-D-Ala C-terminus of the pentapeptide, thus blocking the addition of late precursors by transglycosylation to the nascent peptidoglycan chain and preventing subsequent cross-linking by transpeptidation [10]. Vancomycin has traditionally been reserved as a drug of “last resort,” used only after treatment with other antibiotics had failed [11].

Vancomycin must be given intravenously (IV) for systemic therapy, since it is not absorbed from the intestine. It is a large hydrophilic molecule that partitions poorly across the gastrointestinal mucosa. The only indication for oral vancomycin therapy is in the treatment of pseudomembranous colitis, where it must be given orally to reach the site of infection in the colon [12].

The mean elimination half-life of vancomycin from plasma is 4 to 6 hours in subjects with normal renal function. About 75% of vancomycin is excreted in urine by glomerular filtration in the first 24 hours of its administration. Renal dysfunction slows excretion of the drug resulting in an average elimination half-life of 7.5 days. Therefore vancomycin should be used with care in anephric patients due to its nephrotoxicity which increased by high blood concentration or prolonged therapy [13].

Natural polysaccharides are now extensively used for the development of solid dosage forms for colon drug delivery. A large number of polysaccharides have already been studied for their potential as colon-specific drug carrier systems, such as chitosan, pectin, chondroitin sulphate, cyclodextrin, dextrans, guar gum, inulin, amylose, and locust bean gum [14]. Guar gum and pectin are reported to be potential carriers for colon-specific drug delivery. These studies have shown the drug release retarding property of guar gum in the upper GIT and its degradation by the anaerobic bacteria in the colon [15, 16].

It has been reported that colon-specific delivery system of vancomycin HCl based on pectin hydrogels was developed. This study suggested that pectin/chitosan microspheres were able to limit the release of vancomycin under acidic conditions and release it under simulated colonic conditions, confirming their potential for a colon-specific drug delivery system [17].

In 2008, Bigucci et al. prepared pH-dependent drug release system based on chitosan salts for vancomycin hydrochloride. This study focused on the *in vitro* influence of chitosan salts on the release behavior of vancomycin hydrochloride from the uncoated and coated systems at pH levels of 2.0, 5.5, and 7.6 [18].

Moreover, the influence of polyelectrolyte complexes composed of chitosan and pectin on the release behaviour of vancomycin has been investigated. The precipitated polyelectrolyte complexes were collected by spray-drying. Chitosan/pectin complexes showed a pH-sensitive swelling ability and drug release behavior suggesting their possible use for colon-specific localization of vancomycin [19].

Another study described a controlled drug release system based on chitosan salts for vancomycin hydrochloride delivery. Chitosan aspartate [CH-Asp], chitosan glutamate [CH-Glu], and chitosan hydrochloride [CH-HCl] were prepared by freeze-drying and coated with stearic, palmitic, myristic, and lauric acids by spray-drying technique. The study evaluated, *in vitro*, the influence of chitosan salts on the release behaviour of vancomycin hydrochloride from the freeze-dried and spray-dried systems at pH 2.0 and 7.4 [20].

Vancomycin HCl capsules (125 and 250 mg) are present in the market as oral dosage form intended only for local treatment of pseudomembranous colitis. Being a peptide vancomycin HCl molecules will degrade in the upper part of the GIT due to the premature release of drug in the stomach and small intestine.

Therefore, the present study is an approach to develop an appropriate sustained release colon target drug delivery tablets of this drug which would minimize its inactivation in the upper part of the gastrointestinal tract. These delayed release tablets are designed to improve the efficacy of the drug by concentrating the drug molecules where they are absorbed and thus would ensure lower dosing and less systemic side effects. In addition, CDDS of vancomycin HCl is suggested to be given to patients with renal deficiency to avoid nephrotoxicity. The gradual release of the drug from the sustained release tablets would prevent the accumulation of the drug.

Vancomycin HCl matrix and enteric-coated tablets based on natural polysaccharide, namely, guar gum as a carrier, were formulated. Tablets matrix containing different concentrations of guar gum was prepared by direct compression method and subjected to *in vitro* release studies to find out the efficacy of guar gum in providing sustained release of the drug in the colon. Various release retarding synthetic and natural polymers, namely, hydrogenated castor oil, hydroxypropyl methyl cellulose, xanthan gum, ethyl cellulose, and Eudragit RL 100, were incorporated to modify the drug release rate from the guar gum matrix tablets. Matrix tablets were enteric coated with hydroxypropyl methyl cellulose phthalate as an enteric polymer. Microbiological assays were performed to test the efficacy of selected formulations that showed promising sustained drug release on staphylococcal growth inhibition.

## 2. Experimental

### 2.1. Materials

Materials used are vancomycin HCl (VCM HCl, Mylan, Morgantown, USA), xanthan gum (XG) and guar gum (GG) (Premcem gums Ltd, Mumbai, India), microcrystalline cellulose (Avicel PH 102; FMC Co., Philadelphia, USA), hydrogenated castor oil (HCO; GIRNAR INDUSTRIES, Gujarat, India), hydroxypropyl methyl cellulose (HPMC) and ethylcellulose (EC) (Colorcon, Dartford, UK), hydroxypropylmethylcellulose phthalate (HPMCP; Dow Chemical Co., Michigan, USA), Eudragit RL 100 (Eud. RL 100; Rhon-Klinikum AG-Pharmaceuticals, Bavaria, Germany), and talc and magnesium stearate (BDH Chemicals Ltd; Poole, UK). All other chemical were of reagent grades.

### 2.2. IR Study

IR spectra of physical mixtures (1 : 1) of VCM HCl and various excipients, as well as the drug alone, were performed to find out any possible drug-excipients interaction by KBr pellet method using Perkin-Elmer FTIR series (model-1615) spectrophotometer between 4000 and 450 cm^−1^.

### 2.3. Preparation of Different Matrix Tablets

Vancomycin HCl sustained release tablets were prepared by direct compression technique using guar gum as the main matrix forming materials in different concentrations: 20, 30, 40, 50, and 60% w/w of tablet (300 mg). Other matrix forming materials, namely, HCO, HPMC, EC, Eud RL100, and XG, were added to the guar gum matrix to modulate the drug release. All formulations are listed in Table 1. The calculated amount of the drug and of each ingredient in the formulation was mixed thoroughly by geometric addition in a mortar and then compressed using single punch tablet machine (Erweka, Germany) using 10 mm flat punch under a pressure of 10 Kg. Enteric coating of the prepared matrix tablets was performed using 10% w/v solution of HPMCP in acetone: water (95 : 5 v/v) mixture by dipping methods (15 coats were applied).

### 2.4. Evaluation of Fabricated Matrix Tablets

All prepared matrix tablets were evaluated for its hardness, friability, drug content, and thickness according to official methods [21].

### 2.5. *In Vitro* Dissolution Study

*In vitro* dissolution study of all tablet formulations was carried out using USP apparatus I (Erweka, Germany). The test was performed in 500 mL of 0.1 N HCl for 2 hours then in phosphate buffer pH 6.8 at a temperature of 37 ± 0.5°C. The stirring speed was kept constant at 100 rpm. 5 mL samples were withdrawn at predetermined time intervals of 2, 3, 4, 5, 6, 8, and 24 hours and replaced with preheated fresh dissolution medium. The samples were assayed spectrophotometrically at *λ*_max_ of 281 nm for drug content. All the dissolution tests were run in triplicate and the mean values ± standard deviation (SD) of the percentage cumulative drug release were plotted against time. The results were statistically analyzed using two-way analysis of variance (ANOVA) tests with Tukey's multiple comparison post hoc (Graphbad prism 6 program) to test the significance at a 5% significance level. Statistical difference dealing (*P* < 0.05) was considered.

Excipients used in this study did not interfere in the spectrophotometric reading.

### 2.6. Kinetic Analysis of Dissolution Data

To analyze the *in vitro *release data various kinetic models were used to describe the release kinetics [22]. The zero-order rate (1) describes the systems where the drug release rate is independent of its concentration. The first order (2) describes the release from system where release rate is concentration dependent. Higuchi's model described the release of drugs from insoluble matrix as a square root of time-dependent process based on Fickian diffusion equation (3):

(1) $$\begin{array}{c} C=K_{0}t, \end{array}$$

where *K*_0_ is zero-order rate constant expressed in units of concentration/time and *t* is the time. Consider

(2) $$\begin{array}{c} \log C=\log C_{0}-K_{1}\frac{t}{2.303}, \end{array}$$

where *C*_0_ is the initial concentration of drug and *K*_1_ is first order constant. Consider

(3) $$\begin{array}{c} Q={K_{H}t}^{1/2}, \end{array}$$

where *K*_*H*_ is the constant reflecting the design variables of the system. Consider

(4) $$\begin{array}{c} Q_{0}^{1/3}-Q_{t}^{1/3}=K_{HC}t, \end{array}$$

where *Q*_*t*_ is the amount of drug remained in time *t*, *Q*_0_ is the initial amount of the drug in tablet, and *K*_*HC*_ *t* is the rate constant for Hixson-Crowell rate equation.

### 2.7. Mechanism of Drug Release

Korsmeyer et al. [22] derived a simple relationship which described drug release from a polymeric system equation (5). To find out the mechanism of drug release, first 60% drug release data was fitted in Korsmeyer-Peppas model:

(5) $$\begin{array}{c} \frac{M_{t}}{M_{\infty }}=K\;t^{n}, \end{array}$$

where *M*_*t*_/*M*_*∞*_ is fraction of drug released at time *t*, *K* is the release rate constant incorporating structural and geometric characteristics of the tablet, and *n* is the release exponent. The *n* value is used to characterize different release mechanisms [22, 23].

A plot of log cumulative % drug release versus log time was made. Slope of the line was *n*. The *n* value is used to characterize different release mechanisms as given in Table 2, for the cylindrical shaped matrices. Case II generally refers to the erosion of the polymeric chain and anomalous transport. Non-Fickian refers to a combination of both diffusion and erosion controlled-drug release.

### 2.8. Mean Dissolution Time

Due to the difference in drug release kinetics, the constant *k*, though one of the measures of release rate, should not be used for comparison. Therefore, to characterize the drug release rates in different experimental conditions, another dissolution parameter used for comparing the formulations was mean dissolution time (MDT). This is calculated from the amount of drug released to the total cumulative drug. MDT is a measure of the rate of the dissolution process: the higher the MDT, the slower the release rate. The following equation was used to calculate the MDT from the mean dissolution data [24, 25]:

(6) $$\begin{array}{c} \text{MDT}=\frac{\sum_{i=t}^{i=n}t_{\text{mid}}\;\Delta M}{\sum_{i=t}^{i=n}\Delta M}, \end{array}$$

where *i* is the dissolution sample number, *n* is the number of dissolution sample time, *t*_mid_ is the time at the midpoint between *i* and *i* − 1, and Δ*M* is the additional amount of drug dissolved between *i* and *i* − 1.

### 2.9. Microbiological Study

#### 2.9.1. Staphylococcal Isolates

Three *Staphylococcus aureus* (SA) clinical isolates were studied in this research (SA 11, SA 18, and SA 29). The strains were collected from blood specimens from the outpatient departments of the King Khalid Hospital Riyadh, KSA.

#### 2.9.2. Media and Culture Conditions

All clinical samples were first inoculated onto Sheep Blood Agar (SPML Co. LTD, Riyadh, KSA) and MacConkey Agar (Oxoid, Hampshire, UK) plates. The plates were incubated at 37°C for 24–48 h. The identification of isolates was done according to standard method described by the CDC [26] and Clinical Laboratory Standards Institute [27]. All isolates were stored in brain heart infusion broth containing 16% (w/v) glycerol at −80°C until further use.

#### 2.9.3. Growth on Mannitol Salt Agar

All staphylococcal isolates were reinoculated onto Mannitol Salt Agar (Oxoid, Hampshire, UK) and plates were incubated at 37°C for 24–48 h. Mannitol fermentation was observed and recorded.

#### 2.9.4. Determination of Minimum Inhibitory Concentration (MIC)

MIC of oxacillin (oxacillin sodium monohydrate, Sigma-Aldrich, St. Louis, MO, USA), cefoxitin (cefoxitin sodium salt, Sigma-Aldrich, St. Louis, MO, USA), and vancomycin (vancomycin hydrochloride, Mylan, Morgantown, USA) was determined by agar dilution method as described elsewhere [28]. Briefly, gradient plates of Mueller-Hinton agar (Oxoid, Hampshire, UK) were prepared with oxacillin (0.25–256 *µ*g/mL) (with 2% NaCl), cefoxitin (0.25–256 *µ*g/mL), and vancomycin (0.5–128 *µ*g/mL). By direct colony suspension method, 0.5 McFarland equivalent inoculum was prepared in normal saline from 18 to 24 h agar plate culture. The suspension was further diluted to achieve desired inoculums concentration of 10^5^ CFU/mL. All strains were spotted onto gradient plates. Plates were incubated overnight at 35°C for any visible growth. Readings were taken according to CLSI guidelines.

#### 2.9.5. Time-Kill Curves

The ability of different tablet formulations containing equal amount of vancomycin to inhibit each of the three *Staphylococcus aureus* strains under test was evaluated based on the plotting of time-death curves by an adaptation of the recommendations of the CLSI [29]. A bacterial suspension was prepared from an overnight broth culture, then transferred into Mueller-Hinton broth (Oxoid, Hampshire, UK), and incubated for 2 h at 37°C on a 150 rpm shaking water-bath to reach logarithmic phase. This culture was further diluted in 500 mL phosphate buffer saline pH 6.8 in dissolution flasks using USP apparatus I to achieve an initial bacterial inoculum of ca. 5 × 10^8^ colony-forming units (CFU)/mL or 0.5 McFarland turbidity. Each tablet formulation was challenged against one of the strains tested. The reaction assay was prepared in the dissolution flask in order to mimic the same release pattern of the tablets in the dissolution experiment. The concentration of vancomycin was 100 mg in each tablet. Aliquots of 10 mL of bacterial culture in each dissolution flask were taken at 0, 2, 4, 6, 8, 12, and 24 h of incubation at 37°C, replacing the same amount with fresh buffer. The samples were filtered through Millipore Filter 0.22 *µ*m (Millipore Corporation, Billerica, MA, USA) to control vancomycin carryover and then resuspended in 10 mL sterile saline. The resuspended bacteria were then serially diluted 1 : 10 in sterile saline and 20 *µ*L aliquot was plated on to Mueller-Hinton agar for colony counts. All time-kill curve experiments were conducted in duplicate. Parallel controls were carried out using vancomycin free tablet (Placebo).

#### 2.9.6. Analysis

Mean colony count data (log CFU/mL) were plotted as a function of time for each isolate at each tablet formulation tested. Vancomycin bactericidal effect was defined as ≥99.9% growth inhibition in colony count compared with the starting inoculum count [30].

## 3. Results and Discussion

Since guar gum can be compressed directly in the presence of directly compressible materials such as Avicel, VCM tablets were prepared by direct compression technique for its simplicity.

### 3.1. IR Spectroscopic Studies

**Figure 1:**
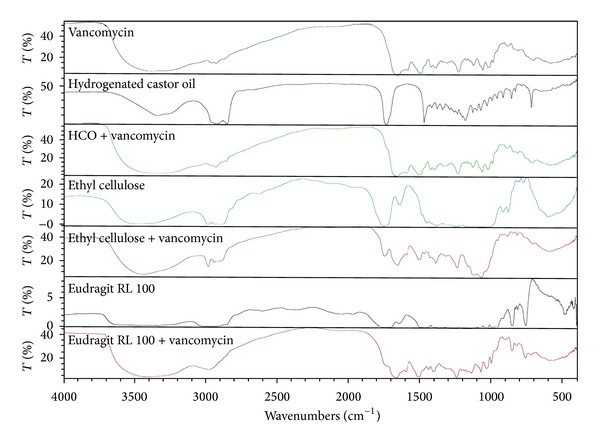
IR spectra of vancomycin HCl, HCO, EC, Eudragit RL 100, and 1 : 1 physical mixtures of the drug and each excipient.

**Figure 2:**
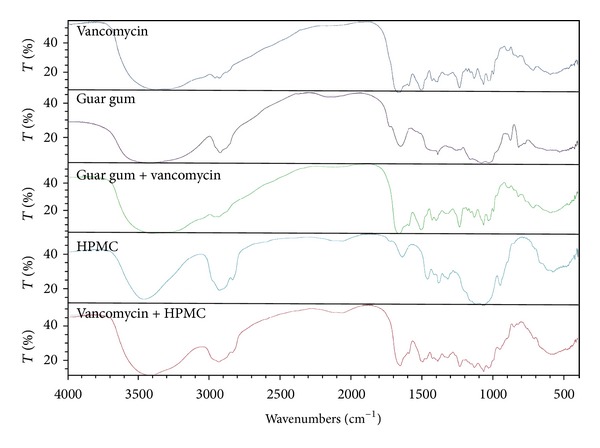
IR spectra of vancomycin HCl, GG, HPMC, and 1 : 1 physical mixtures of the drug and each excipient.

The characteristics spectral bands of pure VCM HCl are stretching of phenolic OH, aromatic C=C, C=O associated with secondary amide function, C–O due to phenolic OH group, Ar–O–Ar and OH deformation at wavenumber of 3401.18, 1651.87, 1505.44, 1396.30, 1231.41, and 1061.54 cm^−1^, respectively [31]. IR spectra of pure drug and physical mixtures of the drug and different excipients are shown in Figures 1 and 2. All the characteristics spectral bands of the drug were not significantly affected in the physical mixture of the drug and excipients. They were retained at their respective positions in the IR spectra of drug-excipient physical mixtures. No significant shift in the position of the characteristics bands was observed indicating absence of interaction between vancomycin HCl and the selected tablet excipients in the physical mixtures.

### 3.2. Evaluation of Physicochemical Parameters of Prepared Tablets

The physical properties of different batches of developed matrix tablets were studied. The thickness of the tablets ranged from 5.20 to 5.40 mm. The hardness of the tablets of all the formulations ranged from 8.3 ± 0.6 to 9.9 ± 0.1 kg/cm^2^. Friability test indicated that the percentage loss was less than 1% (0.53 ± 0.00 to 0.88 ± 0.026). The results of hardness and friability tests denoted that the tablets were hard enough to withstand tablet handling during the study. Drug content was in the range of 99.02 ± 1.0–99.60 ± 0.49%. Weight variation before coating ranged from 297.7 ± 1.85 to 299 ± 1.05 mg while after coating ranged from 357.6 ± 1.62 to 359 ± 1.49 mg.

### 3.3. *In Vitro* Release Study

The present study was aimed at developing novel matrix tablet of vancomycin HCl for colon targeting using guar gum as a matrixing agent. The release of drug depends not only on the nature of matrix but also upon the drug polymer ratio. The percentage of drug released from guar gum matrix tablets reduced in the physiological environment of stomach and small intestine. Majority of drug was released in the physiological environment of colon.

When matrices containing swellable polymers are exposed to dissolution medium, tablet surface becomes wet and hydrated to form a gel layer. The initial release of drug from these matrices occurs by the drug dissolution in the water penetrated into the matrix. The overall drug release from these matrices is governed by hydration, gel layer formation, and drug diffusion into the gel layer and to the dissolution media [32, 33]. Polymer erosion also plays a major role in releasing drug from these matrices [34]. These considerations indicate that hydrophilic polymers have the potential to sustain the release of drug from matrix tablets.

**Figure 3:**
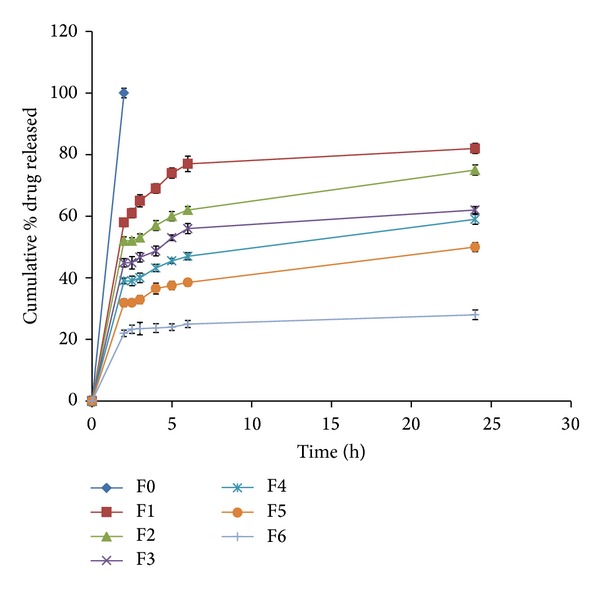
Release profiles of VCM HCl from formulations F0 to F6 containing different concentrations of guar gum (0 to 60% w/w).

**Figure 4:**
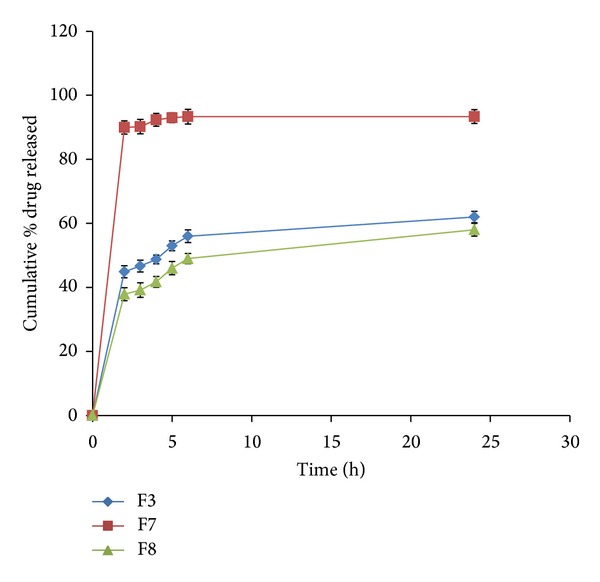
Release profiles of VCM HCl from coated tablet formulations F3 (guar gum matrix), F7 (xanthan gum matrix), and F8 (containing mixture of guar and xanthan gums 1 : 1).

**Figure 5:**
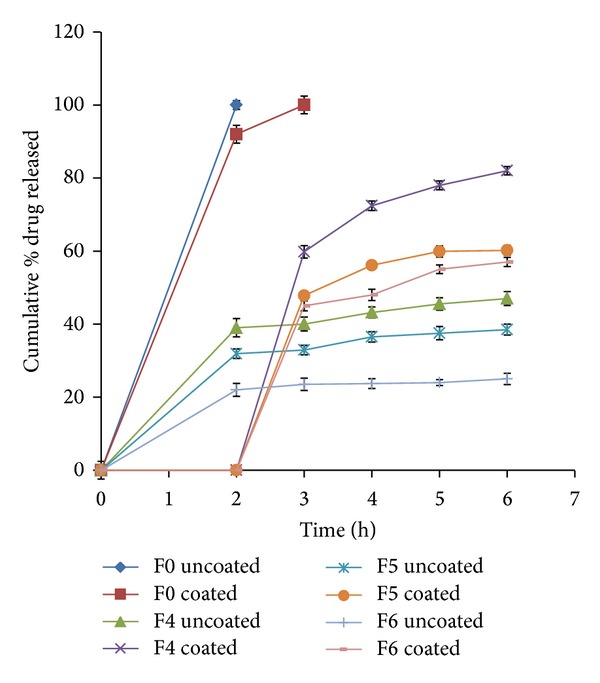
Release profiles of VCM HCl from coated and uncoated F0, F4–F6 containing different concentrations of guar gum (0 to 60% w/w).

**Figure 6:**
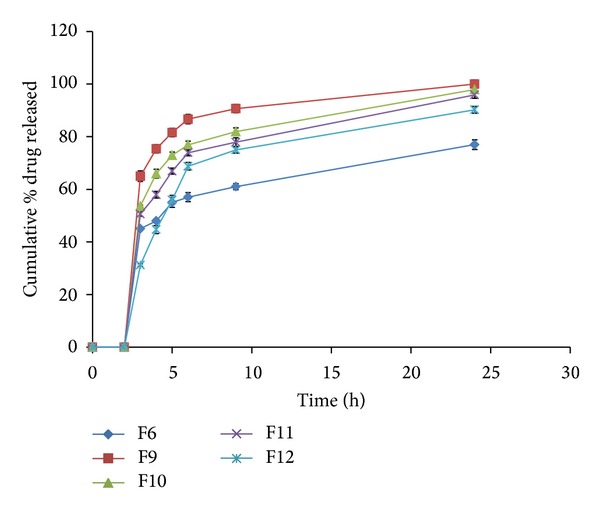
Release profiles of VCM HCl from coated tablet formulations F6, F9–F12 containing different ratios of guar gum and HPMC.

**Figure 7:**
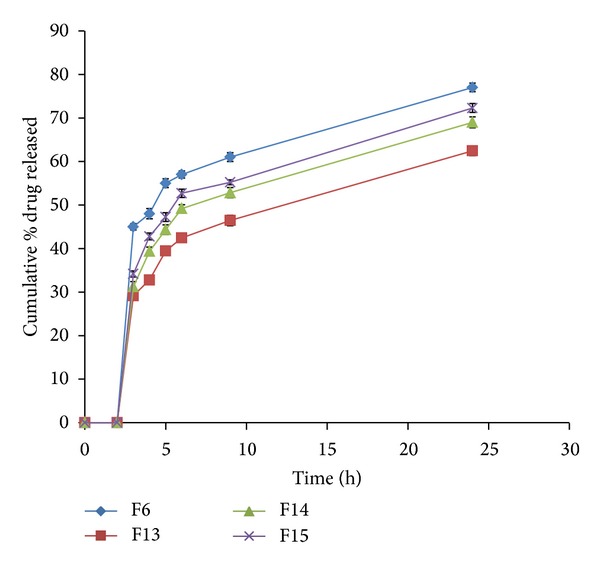
Release profiles of VCM HCl from coated tablet formulations F6, F13–F15 containing different ratios of guar gum and hydrogenated castor oil.

**Figure 8:**
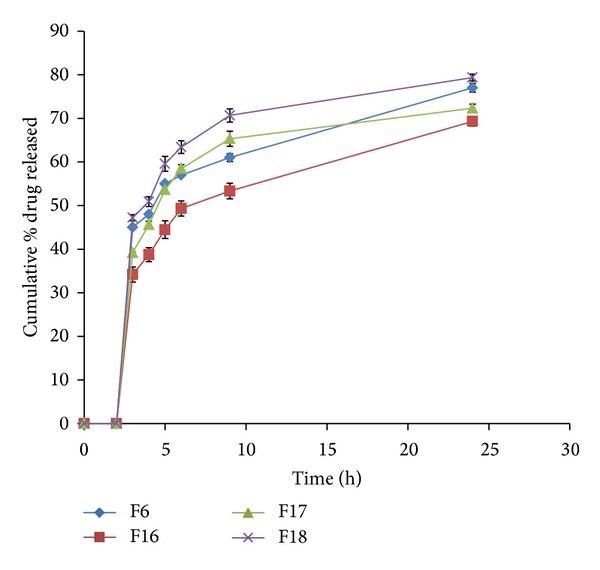
Release profiles of VCM HCl from coated tablet formulations F6, F16–F18 containing different ratios of guar gum and EC.

**Figure 9:**
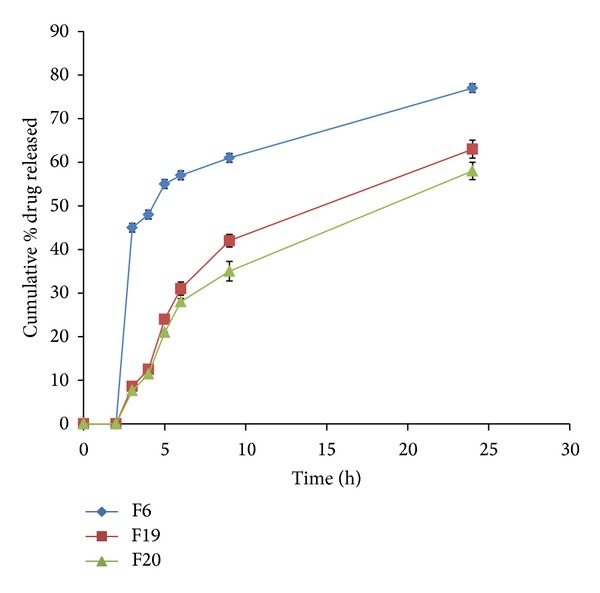
Release profiles of VCM HCl from coated tablet formulations F6, F19, and F20 containing different ratios of guar gum and Eudragit RL 100.

During the dissolution process, a general trend was observed in all the formulations; that is, an increase in polymer concentration resulted in the reduction in amount of drug released. An increase in the polymer concentration causes increase in viscosity of the gel as well as the formulation of gel layer with longer diffusional path. This could cause a decrease in effective diffusion coefficient of drug and therefore a reduction in drug release rate. The *in vitro* cumulative percent drug released versus time profiles of all the tablet formulations is shown in Figures 3, 4, 5, 6, 7, 8, and 9.

The release profiles of formulations F0 to F6 are presented in Figure 3. All formulations released their drug content in acidic pH to various extents depending on guar gum concentration. Increasing the guar gum concentration from 20% w/w (F1) to 60% w/w (F6) reduced significantly the drug release in pH 1.2 from 58% to 22%, respectively, after 2 hrs (*P* < 0.0001). The obtained results can be explained as when the guar gum matrix tablets of vancomycin hydrochloride come into contact with the dissolution medium, they take up water and swell, forming a gel layer around the matrix. Then the dissolved drug diffuses out of the swollen guar gum matrix at a rate determined by the amount and viscosity of guar gum in the tablet formulation [23–25, 31, 32]. Sustained release was displayed by all formulations in phosphate buffer (pH 6.8). The percentage of drug release at the end of 24 hrs of dissolution test (pH 6.8) ranged from 82% (F1) to 28% (F6) with *P* < 0.0001. The release retarding, matrix forming gum was succeeded to sustain drug release over a period of 24 hrs.

Formulations F3, F7, and F8 were prepared to study the influence of the hydrophilic xanthan gum on the release of the water soluble VCM. F3 and F7 matrix tablets contained 33.33% w/w of guar gum or xanthan gum, respectively (Figure 4), whereas F8 containing guar and xanthan gums in the ratio of 1 : 1 was prepared to examine the synergistic or antagonistic effect of both polymers on drug release. Formulation F7 released about 89.96% of its drug content, whereas F3 released 44.89% of the drug at the end of 2 hrs of drug dissolution test (*P* < 0.0001). Since XG is present predominantly in an unionized state at low pH, this results in absence of charged molecules which prevented hydration. Consequently, intermolecular and intramolecular attraction were suppressed leading to inhibition of xanthan hydrogel network formation. Thus increased drug release in pH 1.2 could be explained by the prevention of gel formation of xanthan gum.

Guar gum gives pH-independent drug release due to its nonionic nature. it is not affected by ionic strength or pH [35]. GG had the potential as a release retardant for the water soluble drug than xanthan gum due to gel formation [36]. In addition the release profile of F8 denoted the synergistic effect of the two gums. This could be attributed to the stronger hydrogen bonding between the carboxyl groups of the xanthan and the hydroxyl groups of guar gum, leading to stronger physical cross-linking between the polymers [37].

To overcome the problem of drug release in the acidic pH, the tablet matrix was coated with the enteric polymer HPMCP.  Figure 5 demonstrates the release profiles of coated and uncoated tablet matrices of F4, F5, and F6. It can be noticed that the release of the VCM HCl from coated tablets was completely blocked in pH 1.2 followed by its faster release in pH 6.8 compared to the uncoated tablets (Figure 5). These formulations were selected for further studies because they showed the slowest drug *in vitro* release rates.

Drug release rate could be expected to increase *in vivo* as a result of biodegradation of guar gum by the bacteria present in colon. Many studies reported that the drug release in rat cecal content could be increased to the two or fourfold of its value in presence of the colon bacteria [38, 39]. Based on this consideration, formulation for colon target that showed the slowest drug release *in vitro* would show a reasonable sustained release *in vivo*. Therefore formulation F6 was used for further study. Although coated tablet matrix of formulation F6 succeeded to sustain drug release over a period of 24 hrs, yet it failed to comply with the USP official limits of sustained drug release.

The drug release rate was above the official limits at the specified time intervals. After 1 and 4 hours of dissolution test, the sustained release matrix released 45% and 48% of its drug content, respectively. These drug release percentages were above the official limits which are not more than 25% and 40% after 1 and 4 hours of dissolution test, respectively. Therefore, combination of release retarding polymer (guar gum) and release modifying agents in the formulation of matrix tablets was recommended to modulate the drug release from 60% w/w guar matrix tablet (F6). All the tablets containing different concentrations of each release modifier (F9–F18) were coated with HPMCP polymer to prevent the release of the drug in acidic pH.

HPMC is a hydrophilic cellulose ether, which is used as a retarding polymer in swellable matrices [40].  Figure 6 shows the drug release from guar matrix tablets containing different concentrations of HPMC K4M. It is evident that, in pH 6.8 as the HMPC concentration increased from 7.3 to 20% w/w in F9 to F11, respectively, the drug release extent decreased significantly (*P* < 0.0001) due to faster water absorption capacities. The high water absorption capacities led to a more rapid swelling resulting in the formation of a gel layer with a longer diffusion path and high gel strength which could cause a decrease in the diffusion coefficient of the drug. Therefore a reduction in the drug release was observed.

Matrix tablet formulations F9, F10, and F12 containing 7.3, 14.7, and 22% of HPMC, respectively, showed a faster drug release from 2 to 3 hrs of the release experiment, followed by a slower release from 3 to 24 hrs. Such a biphasic release pattern may be beneficial in providing the initial therapeutically effective plasma concentration followed by an extended plasma concentration. The drug present on the surface of the matrix tablet might have resulted in the initial fast release of the water soluble drug VCM HCl from the formulation. In addition the faster water uptake by HPMC polymer on the surface, leading to formation of loose gel which eroded quickly and increase the diffusion coefficient of the drug from the guar matrix tablets. When the HPMC gel layer on the surface of the tablet eroded, the porosity of tablet increased and facilitated the access of further penetration of the dissolution medium within the tablet [41]. Thus the presence of low concentration of HPMC K4M (7.3% w/w) increased the drug release rate compared to F6. Further increase in concentration of HPMC to 14.7 and 20% w/w in F10 and F11, respectively, reduced the drug release rate compared to F9 (*P* < 0.05) but still higher than F6 (*P* < 0.05). Increasing the concentration of polymer to 30%w/w resulted in a slower drug release rate compared to F6 at the initial stage of dissolution test up to 5 hours, and then the drug release was increased exceeding that from F6 (*P* < 0.05). This can be explained by the following: at the initial stage of dissolution test, in presence of high concentration of HPMC, the fast water uptake capacity leads to rapid formation of a strong gel layer with a longer diffusion path which could cause a reduction in the drug release [41]. Thus HPMC acted as a synergistic gel forming agent which increased the drug release retarding effect of guar gum up to 5 hrs. Then the loose gel of HPMC underwent faster erosion than that of guar gum leading to increased diffusion coefficient and drug release rate.

The effect of hydrogenated castor oil on the drug release from the guar matrix tablet (F13 to F15) is shown in Figure 7. HCO is extremely hydrophobic in nature with lower wettability. It is obvious that increasing the concentration of the hydrophobic polymer in the guar based matrix tablets resulted in a significant decrease in the drug release rate. The hydrophobic nature of the HCO decreased the wettability of the tablet and thus decreased the release of drug present on the tablet surface [42]. In addition HCO being hydrophobic acted as a barrier to water penetration into the tablets, leading to retardation in water absorption, swelling, hydration, and gel formation by guar gum. The extent of retardation in gel formation depended on the concentration of HCO. The release profiles (Figure 7) indicated that increasing the concentration of HCO from 0 to 30% w/w, (F6 to F15) respectively, reduced the drug release rate from 45 (F6) to 34.16% (F15) after 3 hrs (*P* < 0.05). However, increasing the concentration of HCO from 15% w/w (F13) to 30% w/w (F15) increased the drug release rate from 29.16 to 34.16%, respectively. This can be explained by the following: as the concentration of HCO increased, the extent of hydrophobicity of the matrix increased, leading to decrease in the rate and intensity of gel formed by guar gum. Thus, the rate and strength of the gel formed in F13 containing the least concentration of HCO (15% w/w) were higher than those in F14 and F15, containing 25 and 30% w/w HCO, respectively.

Similar results were obtained using ethyl cellulose polymer as a drug release modifier (Figure 8). It can be seen that incorporation of EC in guar matrix tablets F16, F17, and F18 resulted in reducing the drug release rate from guar matrix tablet F6. The obtained results could be due to the hydrophobic nature of EC and its erosion characteristics [42]. The decrease in drug release rate may be attributed to the net result of increased hydrophobicity of the matrix and slow erosion of polymeric content of the matrix tablets.

Incorporation of Eudragit RL 100 in the drug-guar gum matrix (F19 and F20) resulted in a significant decrease in the drug release rate (*P* < 0.05) as shown in Figure 9. Eudragit RL 100 is cationic copolymer of methacrylate with quaternary ammonium groups. It is inert resins and insoluble at physiologic pH but have swelling properties. It is compressible and erodible and due to the presence of 10% quaternary ammonium group the Eudragit matrix is permeable [43]. Thus when the matrix tablet was placed in the dissolution medium the presence of Eudragit RL facilitated the permeation of the dissolution medium into the matrix tablet containing guar gum. The gum rapidly hydrated forming a gel layer inside the matrix and on the matrix surface. The hydrogelation of the gum slowed down the drug release rate from the matrix.

Based on drug release rate studies, the polymers used as release modifiers can be arranged, according to their release retarding efficacy, in ascending order as XG < EC < HPMC < HCO < Eudragit RL 100.

### 3.4. Kinetic Studies

The values of the release exponent (*n*), mean dissolution time, zero-order, first-order, Higuchi release models, and time of 70% drug release for different formulations are presented in Table 2. In the present study the release profiles were not linear suggesting that the drug release from the formulations was not zero order that was confirmed by *R* ^2^ values of 0.820 to 0.947. The release did not fit to first-order model that was also ensured by the low *R* ^2^ values of 0.507 to 0.583. Hixon-Crowell model showed *R* ^2^ values in the range of 0.771 to 0.933. It was observed that the *in vitro* release profiles of drug from all these formulations can be best expressed by Higuchi equation as the correlation coefficients showed the higher values (*R* ^2^: 0.883 to 0.982) (Table 2). Higuchi's kinetics explains why the drug diffuses at a comparatively slower rate (0.048–0.100) as the distance for diffusion increases. To confirm the diffusion mechanism the data was fitted into Korsemeyer-Peppas equation. All the formulations showed slope (*n*) values ranging from 0.30 to 0.69. The *n* values for formulation F6 was 0.43 indicating quasi-Fickian diffusion. The other formulations showed *n* values higher than 0.45 indicating anomalous diffusion or non-Fickian diffusion. Anomalous diffusion or non-Fickian diffusion refers to a combination of both diffusion and erosion controlled-drug release.

The release rate and *T*_70%_ values of these formulations can be considered as a function of the type and concentration of the retarding polymer used. The differences in drug release rate and *T*_70%_ among the different formulations are confirmed from their MDT data. MDT value is used to characterize the drug release rate from the different formulation and the retarding efficacy of the polymers. It is obvious that guar gum in 60% w/w concentration showed the higher value of MDT indicating high polymer retarding efficacy. In general, polymers used as release modifiers in this study can be arranged as an efficient polymer based on MDT as HCO > HPMC > EudRL 100 > EC. It was also observed that on using the same modifier MDT values varied according to the concentration and accordingly the ratio between the release retarding and release modifier polymers, for example, in case of HPMC as the concentration increased the MDT increased except for F12 of the highest concentration of polymer, showed the least value of MDT. The similar results were observed in case of EC (Table 2). These observations may be explained by the net mechanism of drug release influenced by guar gum and the modifier type and ratio.

**Figure 10:**
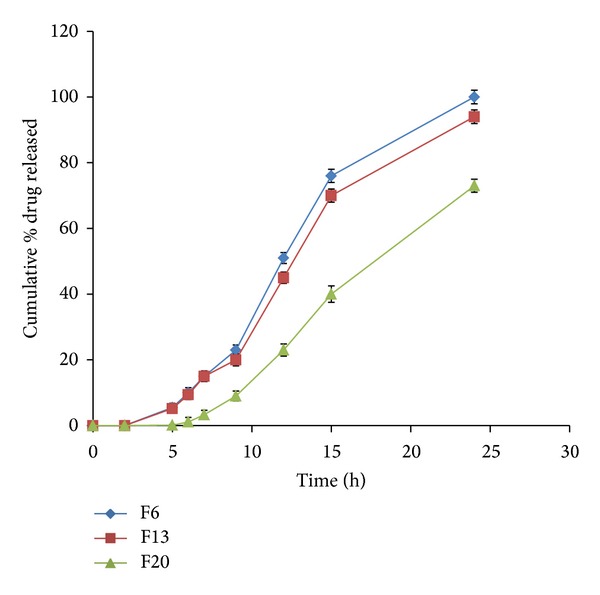
Release profiles of VCM HCl from coated tablet formulations F6, F13, and F20 containing different ratios of guar gum and Eudragit RL 100 in pH 1.2 for 2 hours, in pH 7.4 for further 3 hours, and then in pH 6.8 till the end of 24 hours.

Formulations F6, F13, and F20 were selected for further study depending on their MDT values 8.25, 7.97, and 7.64, respectively. They also showed promising results as sustained release formulations. They were subjected to further examination of the drug release in different pHs along the passage of the formulations through the GIT. The drug release rate was determined in pH 1.2 for 2 hrs followed by pH 7.4 for further 3 hrs then in pH 6.8 up to 24 hrs. The drug release was blocked in pH 1.2 due to HPMCP coating. After 5 hours of the release study, the drug released in pH 7.4 was 5.5, 5.2%, and 0.1% from F6, F13, and F20, respectively (Figure 10). In pH 6.8, formulations F20 showed significant reduction in drug release rate compared to formulations F6 and F13 (*P* < 0.05). The three formulations showed sustained release characteristics over 24 hours. The decrease in drug release rates after 2 hours of dissolution in pH 6.8 (*in vitro*) could be expected to increase by 2- to 4-fold in the presence of rat cecal content [38, 39]. This expectation could lead the formulations to comply with the USP specifications of sustained release rate.

The three selected formulations that showed promising sustained release characteristics (F6, F13, and F20) were further evaluated microbiologically to examine the efficacy of each formulation in inhibiting the growth of *Staphylococcus aureus* clinical isolates.

### 3.5. Microbiological Studies

Formulations F6, F13, and F20 were challenged microbiologically against three strains of *Staphylococcus aureus* 11, 18, and 29. The susceptibility of the three strains was tested against oxacillin, cefoxitin, and vancomycin and the results were obtained in Table 3. According to the recommendations of the CLSI (2011), *Staphylococcus aureus* strains are considered methicillin resistant if they are resistant to both oxacillin and cefoxitin. Strain 18 was identified as methicillin sensitive SA (MSSA) since it is sensitive to both cefoxitin and oxacillin and its MIC against vancomycin was 2 *µ*g/mL. Strains 11 and 29 were identified as methicillin resistant SA (MRSA) since their minimum inhibitory concentration (MIC) values were greater than 4 and 8 *µ*g/mL for oxacillin and cefoxitin, respectively. While MRSA 29 was considered vancomycin sensitive (MIC 2 *µ*g/mL), MRSA 11 was considered vancomycin intermediate (MIC 8 *µ*g/mL). The identification of the strains followed the MICs values reported by CLSI (2011) [9].

**Figure 11:**
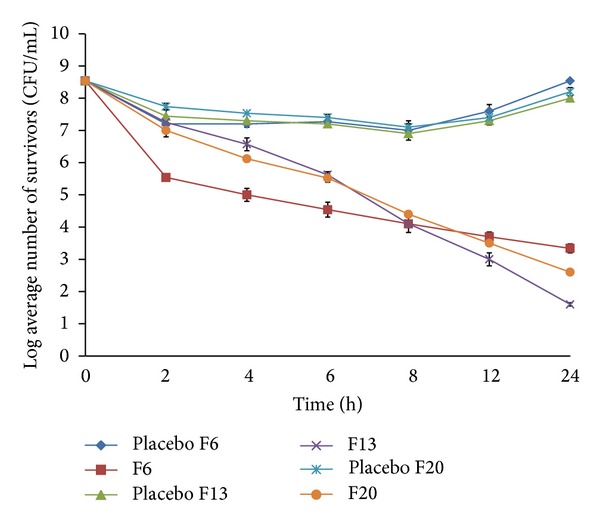
*In vitro* time-kill kinetics against the clinical strain MSSA 18 performed in phosphate buffer pH 6.8 after exposure to uncoated tablet formulations F6, F13, and F20 and their drug-free control. Each data point represents the mean of 2 independent experiments. CFU: colony-forming units.

**Figure 12:**
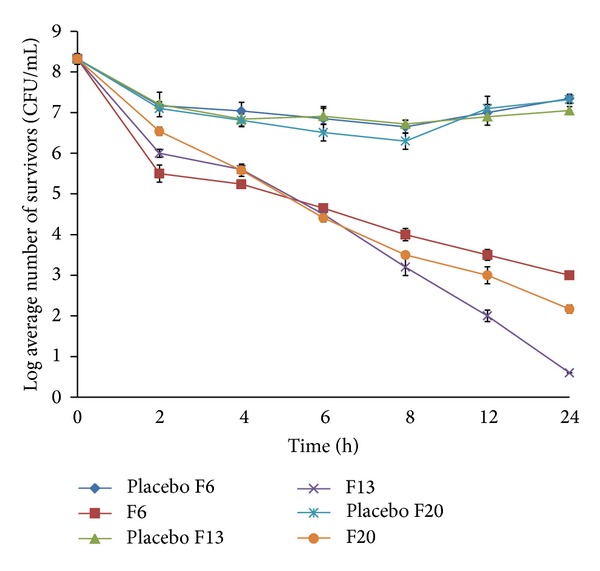
*In vitro* time-kill kinetics against the clinical strain MRSA 29 performed in phosphate buffer pH 6.8 after exposure to uncoated tablet formulations F6, F13, and F20 and their drug-free control. Each data point represents the mean of 2 independent experiments. CFU: colony-forming units.

**Figure 13:**
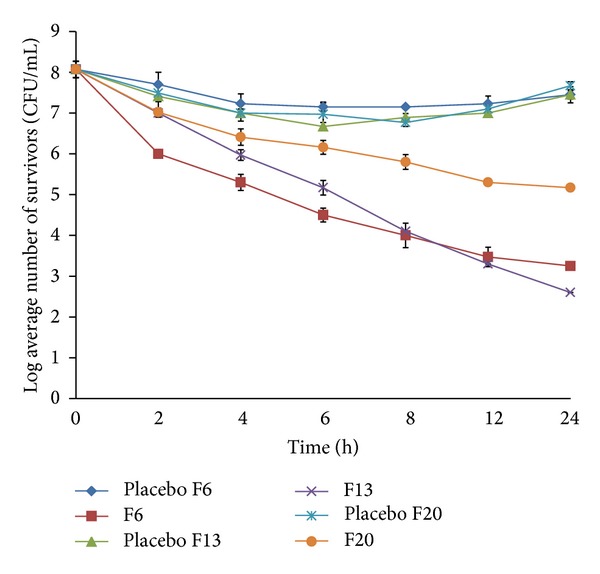
*In vitro* time-kill kinetics against the clinical strain MRSA 11 performed in phosphate buffer pH 6.8 after exposure to uncoated tablet formulations F6, F13, and F20 and their drug-free control. Each data point represents the mean of 2 independent experiments. CFU: colony-forming units.

Time-kill data for the three isolates against the basic formulation F6 and the modified formulations (F13 and F20) were presented in Table 4 and Figures 11, 12, and 13. Generally all three isolates had distinct *in vitro* time-kill activity profiles against the new vancomycin formulations tested. Moreover, control or placebo tablets resulted in minimal kill for all isolates tested with regrowth occurring by 24 hrs.

There was a time difference between the formulas in reaching bactericidal activity (3 log reduction in microbial count) against the three strains tested (Table 4). F6 displayed the fastest bactericidal activity since the formulation was more or less faster in its drug release. The release of vancomycin was about 48% after 2 hours of *in vitro* dissolution study, which was enough to kill 99.9% of MSSA 18 but the same formula needed 4 hours to reach the same effect for the other two MRSA strains. Although there was no significant time difference between the other two modified sustained release formulas (F13 and F20) in reaching bactericidal activity ( 6 and 6–8 hours, resp.) against all strains tested, there was a noticeable difference in their vancomycin release pattern since it was about 42.45% and 28%, respectively. This can be explained by the fact that the hydrogenated castor oil present in F13 being waxy in nature formed a protective layer on the microorganism cell wall against the invasion of the drug and thus may delay the effect of the antibiotic on the bacterial isolates. This protective layer would be eroded by time and inhibitory effect of the drug was manifested. Due to the slow release pattern of the vancomycin from the formulas, exponential killing effect was demonstrated by time especially for F13 which showed much higher biocidal effect after 24 hours of incubation than the other two formulas. Its log microbial inhibition exceeded F6 by 1.74, 0.65, and 2.4 CFU/mL for MSSA 18, MRSA 11, and MRSA 29, respectively, while it was 1, 2.57, and 1.57 CFU/mL for MSSA 18, MRSA 11, and MRSA 29, respectively, compared to F20 (Figures 11–13).

Figure 11 exhibited the time killing results of the three different formulations F6, F13, and F20 against the methicillin sensitive strain MSSA 18. By comparing the vancomycin free formulas together against each other, the results showed no significant differences between the controls of the three formulations since the difference ranged only from 0.1 to 0.5 CFU/mL log reduction in bacterial survival and this can be attributed to the comparable structure of the guar gum with either Eudragit RL 100 or hydrogenated castor oil.

After 6 hours of dissolution experiment, formulation F6 released about 10% and 30% more vancomycin than formulas F13 and F20, respectively. This was better demonstrated by the time-kill assay since F6 resulted in 2.73 log reduction in CFU/mL while it was only 1.58 and 1.88 CFU/mL for F13 and F20, respectively, compared to the placebo of each formulation. On the other hand, F6 showed less inhibitory effect after 24 hrs of incubation than the other two sustained release formulas F13 and F20 whose effect started to be bactericidal from 6 hours onward and showed better bactericidal effect after 24 hours of incubation though F20 appeared to be slightly less active than F13. Compared to the control which is the vancomycin free (placebo) formulations, considerable inhibition occurred after 8 hours of incubation for all formulations tested but after 24 hours of incubation the log reduction in the microbial count was approximately 5.2, 5.6, and 6.4 CFU/mL for F6, F20, and F13, respectively.

Time-kill data for MRSA 29 were displayed in Figure 12. The bacterial strain had distinct *in vitro* time-kill activity profiles against all formulations starting from the beginning of the experiment. However, after 2 hours of incubation, F20 exhibited the minimum killing activity with no more than 0.5 and 1.8 CFU/mL log reduction compared to the placebo and the initial inoculums, respectively. Approximately 3 log reduction in bacterial count was achieved after 6 hours of incubation onwards using any of the formulations tested.

Although there was better killing activity of F6 against the mentioned strain starting from the beginning of the assay, the rate of killing was slower than F13 and F20 which showed more potent killing effect by time causing about 7.7 and 6.2 CFU/mL log microbial reduction after 24 hours, respectively, compared to 5.3 CFU/mL log reduction obtained by F6 after the same time.

Comparing the placebo formulas together, there was no considerable killing activity on the strain tested giving no more than 2 log microbial inhibition with regrowth of the strain by time.

As for MRSA 11 strain which displayed methicillin resistance and vancomycin intermediate susceptibility the time-kill pattern of the strain against the formulations tested was a lot different since the maximum reduction in microbial count reached along the experiment ca. 5 CFU/mL after 24 hours using F13 formula (Figure 13). Furthermore, the isolate displayed the maximum difference between the sustained release formula F20 and the fast release formula F6 since the latter decreased the bacterial count over time and showed a significant better killing effect from the beginning of the experiment onwards reaching the level of bactericidal endpoint (99.9% killing) after ca. 4 hours. Although F13 results against the strain exhibited slightly better reduction in bacterial count after 24 hours, F6 showed higher reduction in log number of bacterial survival for the first several hours till nearly after 8 hours. Again there was indifferent activity between the reactions of the controls which lack vancomycin against MRSA 11.

## 4. Conclusion

Vancomycin is considered to be the drug of last resort in treating systemic staphylococcal infections as 500 mg I.V. injections. In the present study oral tablet formulations containing only 100 mg vancomycin were prepared. The results of this work revealed that among 20 prepared vancomycin tablets, only three formulations showed promising sustained release *in vitro*. The selected tablets showed a great bactericidal effect on either methicillin sensitive or methicillin resistant *Staphylococcus aureus* confirming the efficacy of the chosen formulation and dose. Further trials on experimental animals are recommended.

## Figures and Tables

**Table 1:** Composition of different vancomycin HCl matrix tablets prepared by direct compression.

Formulation code		Amount of ingredients in mg							
	Drug	GG	XG	HPMC	HCO	EC	Eud. RL	Avicel	Mg. st : talc*
F0	100	—	—	—	—	—	—	195	5
F1	100	60	—	—	—	—	—	135	5
F2	100	90	—	—	—	—	—	105	5
F3	100	100	—	—	—	—	—	75	5
F4	100	120	—	—	—	—	—	45	5
F5	100	150	—	—	—	—	—	15	5
F6	100	180	—	—	—	—	—	95	5
F7	100	—	100	—	—	—	—	95	5
F8	100	50	50	—	—	—	—	95	5
F9	100	158	—	22	—	—	—	15	5
F10	100	136	—	44	—	—	—	15	5
F11	100	114	—	66	—	—	—	15	5
F12	100	90	—	90	—	—	—	15	5
F13	100	135	—	—	45	—	—	15	5
F14	100	105	—	—	75	—	—	15	5
F15	100	90	—	—	90	—	—	15	5
F16	100	135	—	—	—	45	—	15	5
F17	100	105	—	—	—	75	—	15	5
F18	100	90	—	—	—	90	—	15	5
F19	100	90	—	—	—	—	90	15	5
F20	100	135	—	—	—	—	45	15	5

*Mixture of magnesium stearate (Mg. st) and talc in the ratio of 2 : 1 was used as a lubricant.

**Table 2:** Results of kinetics study.

Formula	Correlation coefficients (*r* ^2^)				Korsemayer'sslope (*n*)	*T*_70%_ in hour	MDT in hour	DR after 9 hours	DE_9 h_ %
	Zero order	First order	Hixon-Crowell	Higuchi's model					
F6	0.952	0.537	0.933	0.982	0.43	21.82	8.25	61.00	29.71
F9	0.820	0.507	0.833	0.883	0.52	3.72	4.96	90.67	34.00
F10	0.882	0.529	0.847	0.932	0.50	4.80	6.28	81.91	31.36
F11	0.902	0.540	0.870	0.947	0.51	5.68	6.74	77.91	28.16
F12	0.826	0.583	0.771	0.889	0.56	8.40	4.83	74.99	31.16
F13	0.947	0.572	0.917	0.978	0.60	>24	7.97	46.45	27.89
F14	0.923	0.565	0.932	0.962	0.57	>24	7.19	52.82	28.68
F15	0.926	0.558	0.887	0.961	0.56	23.25	7.40	55.21	28.66
F16	0.940	0.559	0.912	0.974	0.60	>24	7.95	53.32	28.85
F17	0.825	0.535	0.795	0.890	0.54	23.23	4.37	65.32	33.87
F18	0.864	0.528	0.839	0.920	0.52	8.92	5.05	70.76	32.97
F19	0.923	0.552	0.841	0.963	0.69	>24	6.80	42.00	25.00
F20	0.940	0.553	0.859	0.945	0.58	>24	7.64	23.00	22.63

**Table 3:** Biostatic activity to different antibiotics against S11, S18, and S29 isolates.

Antibiotic	*S. aureus* strain *MIC (*μ*g/mL)			**Species-related breakpoints	
	S11	S18	S29	*R*	*S*
Oxacillin	>256	2	32	≥4	≤2
Cefoxitin	64	4	16	≥8	≤4
Vancomycin	8	1	2	>16	≤2

*MIC: minimum inhibitory concentration.

**CLSI, 2011.

**Table 4:** Time (h) to achieve bactericidal activity (99.9%) reduction of the initial inoculums.

Strain tested	Formula examined		
	F6	F13	F20
MSSA 18	2	6	6
MRSA 29	4	6	6
MRSA 11	4	6	8
